# A rare case of adult herpes simplex encephalitis complicated with rhabdomyolysis

**DOI:** 10.1186/s12879-021-05798-1

**Published:** 2021-01-23

**Authors:** Qinwei Yu, Chao Han, Lei Pei, Jinsha Huang, Yan Xu, Tao Wang

**Affiliations:** 1 Department of Neurology, Union Hospital, Tongji Medical College, Huazhong University of Science and Technology, 1277 Jiefang Road, Wuhan, 430022 Hubei China; 2 Department of Neurology, Division of Life Sciences and Medicine, the First Affiliated Hospital of USTC, University of Science and Technology of China, Hefei, 230001 Anhui China; 3 Traditional Chinese Medicine Hospital of Gongan County, 234 Youjiang Road, JingZhou, 434300 Hubei China

**Keywords:** Herpes simplex encephalitis, Rhabdomyolysis, Virus, Seizures

## Abstract

**Background:**

Compelling evidence indicates that status epilepticus is a prevalent cause of rhabdomyolysis. However, cases of rhabdomyolysis induced by a single seizure accompanied by viral encephalitis are rarely reported. Herein, we present a case of adult Herpes Simplex Encephalitis complicated with rhabdomyolysis.

**Case presentation:**

A 32-year-old male was patient presented with fever accompanied by episodes of convulsions, myalgia, and oliguria, which exacerbated the delirium. Routine blood examination showed impaired kidney function and elevated myoglobin (Mb) and creatine phosphokinase (CK) levels. MRI scanning revealed a damaged frontotemporal lobe and limbic system. In addition, herpes simplex virus (HSV) pathogen was identified in the cerebrospinal fluid thus indicating HSV infection. Therefore, a diagnosis of rhabdomyolysis triggered by HSV infection accompanied by epilepsy was made. Notably, the patient recovered well after early intervention and treatment.

**Conclusion:**

The case presented here calls for careful analysis of rhabdomyolysis cases with unknown causes, minor seizures, and without status epilepticus. This case also indicates that HSV virus infection might contribute to the rhabdomyolysis.

**Supplementary Information:**

The online version contains supplementary material available at 10.1186/s12879-021-05798-1.

## Background

Rhabdomyolysis is a disease that disrupts the integrity of muscle cell membranes causing unprecedented release of cell contents such as myoglobin (Mb), enzymes like creatine phosphokinase (CK), and toxic substances such as ions into the blood [1]. These factors trigger internal environment disorders, and acute kidney injury or other tissue/organ damage in more serious cases. Rhabdomyolysis is caused by factors including trauma, variation in body temperature, muscle hypoxia, drugs, toxins, electrolyte or metabolic disorders, viral and bacterial infections, as well as the status epilepsy [2]. However, rhabdomyolysis induced by a single seizure is largely unexplored. Notably, Herpes simplex encephalitis (HSE) is a necrotizing encephalitis induced by herpes simplex virus (HSV) infection. Based on its antigenicity, HSV is classified into HSV-1 and HSV-2. Adult HSE is frequently caused by HSV-1 infection in the frontotemporal lobe and limbic system whereas HSV-2 infection induces genital diseases and neonatal encephalitis [3]. Reports indicate that 50% of patients diagnosed with HSE might develop epilepsy [4]. This study report an unusual case of rhabdomyolysis triggered by a single epilepsy, rather than status epilepticus. The condition stemmed from HSV infection.

## Case presentation

**Fig. 1:**
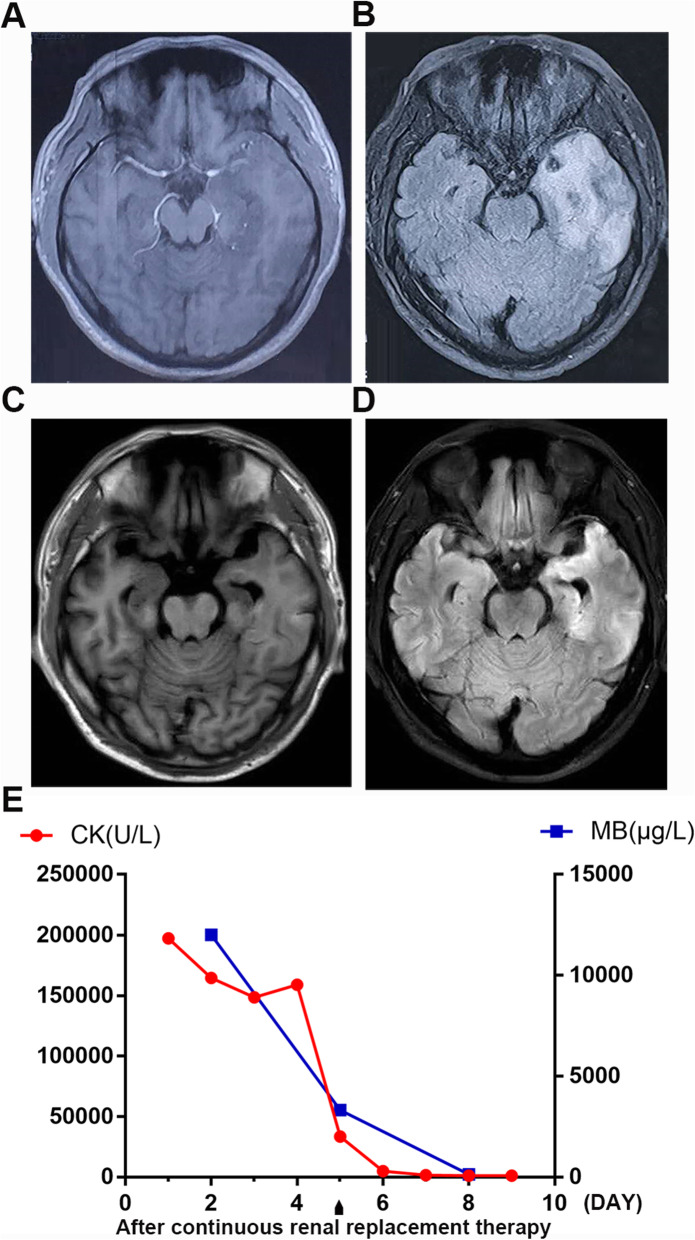
the changes of MRI images and content of MB and CK before and after treatment. **a** and **b** The left temporal lobe, hippocampus and insula were swollen as seen on the MRI image and high T2 signal was seen in these areas upon hospitalization. **c** and **d** MRI images taken at follow-up revealed significant improvement of the intracranial lesions. **e** The trend of Mb and CK of the patient before and after treatment of hemodialysis. Mb, myoglobin. CK, creatine phosphokinase

A 32-year-old male patient with a medical history of hepatitis without poisoning presented to our hospital because of untreated mild fever (not exceeding 38.5 °C) for 5 days and blood urine. Moreover, the patient developed epilepsy accompanied by unconsciousness. Upon hospitalization, he was depressed. His vital signs including blood pressure, heart rate, and oxygen saturation were normal. Neurological examination revealed a mild stiffness in the neck and gastrocnemius pain. Besides, a positive Babinskin sign was detected, and no other abnormal findings were identified. Routine blood examination showed extremely high Mb (12,000 g/L, normal < 140 g/L) and CK (197,159 U/L, normal 38-174 U/L), low GFR (glomerular filtration rate) demonstrated impaired renal function (26 ml/min, normal 90-120 ml/min). White blood cells, CRP (C-reactive protein), ESR (erythrocyte sedimentation rate), hepatic function were moderately elevated. Other indices including the ANCA/GBM, tumor markers and thyroid function, blood sugar, electrolyte, and sexually transmitted diseases (including human immunodeficiency virus (HIV)) were within normal range. Further, cerebrospinal fluid (CSF) examination revealed elevated protein levels (0.92 g/L, normal 0.15–0.45 g/L) and mildly increased leukocytes (12.9*10 ~ 6/L, normal < 8*10 ~ 6/L), indicating a high possibility of viral encephalitis infection. The left temporal lobe, hippocampus, and insula were swollen and exhibited a high T2 signal as observed on the MRI image (Fig. 1a and b). Considering the MRI findings and his clinical manifestation, this study tested for the presence of HSV pathogen in the CSF. As expected, the HSV-1 gene sequence was detected via DNA sequencing of pathogenic microorganisms. Notably, HSV-2 and other common causes of rhabdomyolysis such as varicella-zoster virus, Epstein-Barr virus, cytomegalovirus, human herpesvirus 6, enterovirus, dengue, Zika, West Nile, H1N1 or scrub typhus infection were not detected via the Metagenome Next Generation Sequencing (mNGS). Besides, based on the sequencing results, other bacteria, fungi, parasites, and mycobacterium were negative. Furthermore, serum CMV, enterovirus, and coxsackievirus were negative (Supplementary material 1 and 2). As a result, herpes simplex encephalitis infection was confirmed. Based on these findings, the patient was eventually diagnosed with rhabdomyolysis, acute renal failure, and epilepsy due to HSV infection. During 3 weeks of hospitalization, the patient was antivirally treated with 300 mg acyclovir at intervals of every 8 h a day, antiepileptic management with 300 mg oxcarbazepine, and 500 mg sodium valproate simultaneously administered twice a day. Also, the patient was administered with 10 mg dexamethasone to manage inflammation. Due to the extremely high content of Mb and CK, 1000 ml saline and a single hemodialysis sustained for 8 h were used as rehydration therapy (Fig. 1e). A complete remission was achieved after 3 weeks of treatment. After discharge, the patient was placed on continuous orally antiviral (acyclovir, 100 mg, three times a day) and antiepileptic drugs (sodium valproate, 500 mg, twice a day) for 4 weeks, after which he returned for a follow-up visit. MRI images taken at follow-up revealed significant improvement of intracranial lesions (Fig. 1c and d) and the viral PCR using the CSF sample was negative for HSV-1 and HSV-2.

## Discussion and conclusion

This study presents a case of HSE with secondary epilepsy, accompanied by rhabdomyolysis and acute renal failure. The initial symptoms included fever and headache, followed by convulsions, notably, without dominant signs of herpes simplex infection such as skin lesion. CSF examination and typical images of MRI confirmed the diagnosis of HSE. Besides, based on symptoms such as myalgia, hematuria, and high blood content of CK and Mb, we confirmed rhabdomyolysis and acute renal failure.

Importantly, rhabdomyolysis induced by HSE, a rarely reported complication was a novel finding of the present case. Despite the prevalent cause being status epilepticus, epilepsy triggered by HSE was ascribed to the occurrence of rhabdomyolysis [5]. Given that the patient was highly muscular, it was likely that a single strong contraction of muscles could rupture the muscle cells [6]. Besides, the virus itself might cause rhabdomyolysis, as in the case of influenza virus infection frequently reported frequently [7]. Other causes including herpes simplex virus, coxsackievirus, and HIV have been reported [8]. In the present case, a broad range of coinfection factors including pathogenic microorganisms known to trigger multisystem dysfunction were excluded. In addition, other underlying metabolic factors as among them, hypoglycemia, hypothyroidism, electrolyte disturbance, hypoxia, and food deficiency were ruled out. Concerning the mechanisms, we hypothesized that HSE infection detected in the present case might have originated from direct viral invasion into the muscle cells, attributed to the presence of other reported viral DNA in the muscle specimen [8, 9]. Also, the circulating viral toxins might damage the muscle cells [1]. Nonetheless, the specific mechanisms by which HSV induces rhabdomyolysis are underexplored. Therefore, we postulated that the combined effects of epilepsy and virus infection triggered the occurrence of rhabdomyolysis after HSE infection.

Rhabdomyolysis is relatively rare in clinical practice, with varying etiology. Moreover, its typical symptoms such as myalgia, soy urine, and fatigue are manifested in less than 10% of all patients [10]. The mortality rate of rhabdomyolysis is estimated at 61.2% of all clinically ill patients [11]. Herein, we suggest that timely detection and diagnosis, immediate administration of antiviral and rehydration therapies, is critical for a full recovery.

## Supplementary Information

**Additional file 1.**

**Additional file 2.**

## Data Availability

All data generated or analyzed are included in this published article.

## Abbreviations

Mb: Myoglobin
CK: Creatine phosphokinase
HSV: Herpes simplex virus
HSE: Herpes simplex encephalitis
GFR: Glomerular filtration rate
CRP: C-reactive protein
ESR: Erythrocyte sedimentation rate
ANCA/ GBM: Antineutrophil cytoplasmic antibody/ Glomerular Basement Membrane
CSF: Cerebrospinal fluid
HIV: Human immunodeficiency virus
