# Supplementary material for: A rare case of adult herpes simplex encephalitis complicated with rhabdomyolysis
Source: BMC Infect Dis. 2021 Jan 23;21:110. doi: 10.1186/s12879-021-05798-1 (PMC7825203; doi:10.1186/s12879-021-05798-1)
Supplement: Supplementary file 1 — Additional file 1. [file 12879_2021_5798_MOESM1_ESM.docx]

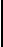
Guangzhou Darui Medical Laboratory


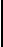


**Pathogenic microorganism test report**

Overview

Infectious diseases are one of the common clinical diseases, mostly pathogens such as bacteria, viruses and fungi and their metabolites. The local or systemic inflammation or organ dysfunction caused by these pathogens exerts greater harm and induces potential high mortality. Fastly and accurately identifying pathogens and choosing a reasonable treatment plan can effectively control the occurrence and development of the disease. While current routine detection methods for pathogens have limitations such as low positive rate, long cycle, and high technical requirements for operators.

mNGS (Metagenome Next Generation Sequencing) is a new term proposed by Handelsman et al. in 1998. It is defined as "the genomes of
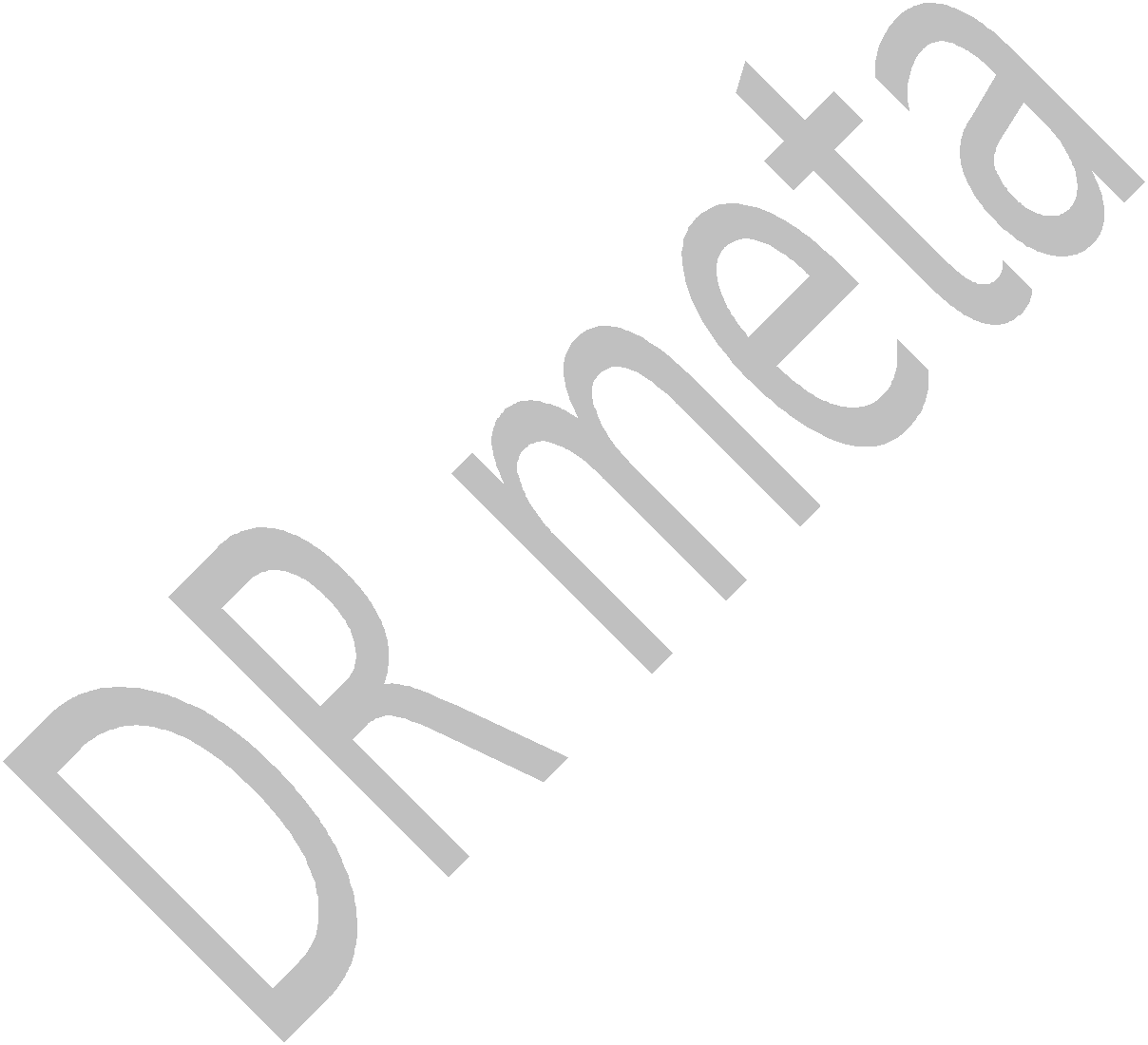
 the total microbiota found in nature", which means the sum of the genetic material of all microorganisms, including all the genomes of cultivable and non-culturable microorganisms. Metagenomics is a kind of research on the microbial population genome in environmental samples, with functional gene screening and sequencing analysis

Metagenomic sequencing refers to the high-throughput sequencing of all pathogen genomes in specific environmental samples. The method can quickly, accurately and efficiently obtain the genome information of the entire pathogen population. Metagenomic sequencing does not rely on the isolation and culture of pathogens, information about pathogens with low abundance and even trace amounts in the environment can be obtained [1-3]. In recent years, Metagenomic sequencing is increasingly used in medical research and clinical diagnosis, such as infection type diagnosis, resistance gene. Identification of causes and prevention and control of infectious diseases, etc [4-9].

DR meta can detect 10635 kinds of microorganisms, including 5456 kinds of bacteria (including 104 kinds of mycobacteria, 45 Species of Mycoplasma/Chlamydia and 25 species of spirochetes), 411 species of fungi, 4599 species of viruses, 1773 species of DNA viruses, RNA 2826 kinds of viruses, 169 kinds of parasites and 4417 kinds of drug resistance genes (31 antibiotics);

The detection process includes: pretreatment, nucleic acid extraction, cDNA synthesis, fragmentation, library construction, sequencing, biological Information analysis and report interpretation, etc.;

DR meta has self-developed databases, including drug resistance gene database, report database and case database.

Guangzhou Darui Medical Laboratory


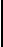


| **2.patient information** | |  |  | Barcode：320190210900 | |
| --- | --- | --- | --- | --- | --- |
|  |  |  |  |  |  |
| Name： | *** | Sex： | man | Age： | 32 |
| Sample acquired time ： | 191112 | Sample collection time： | 191114 | Report time： | 191117 |
| Doctor： |  | sample： CSF | |  |  |
| Department： neurology | |  |  |  |  |
| Hospital：Wuhan Union hospital | |  |  |  |  |
|  | | |  |  |  |


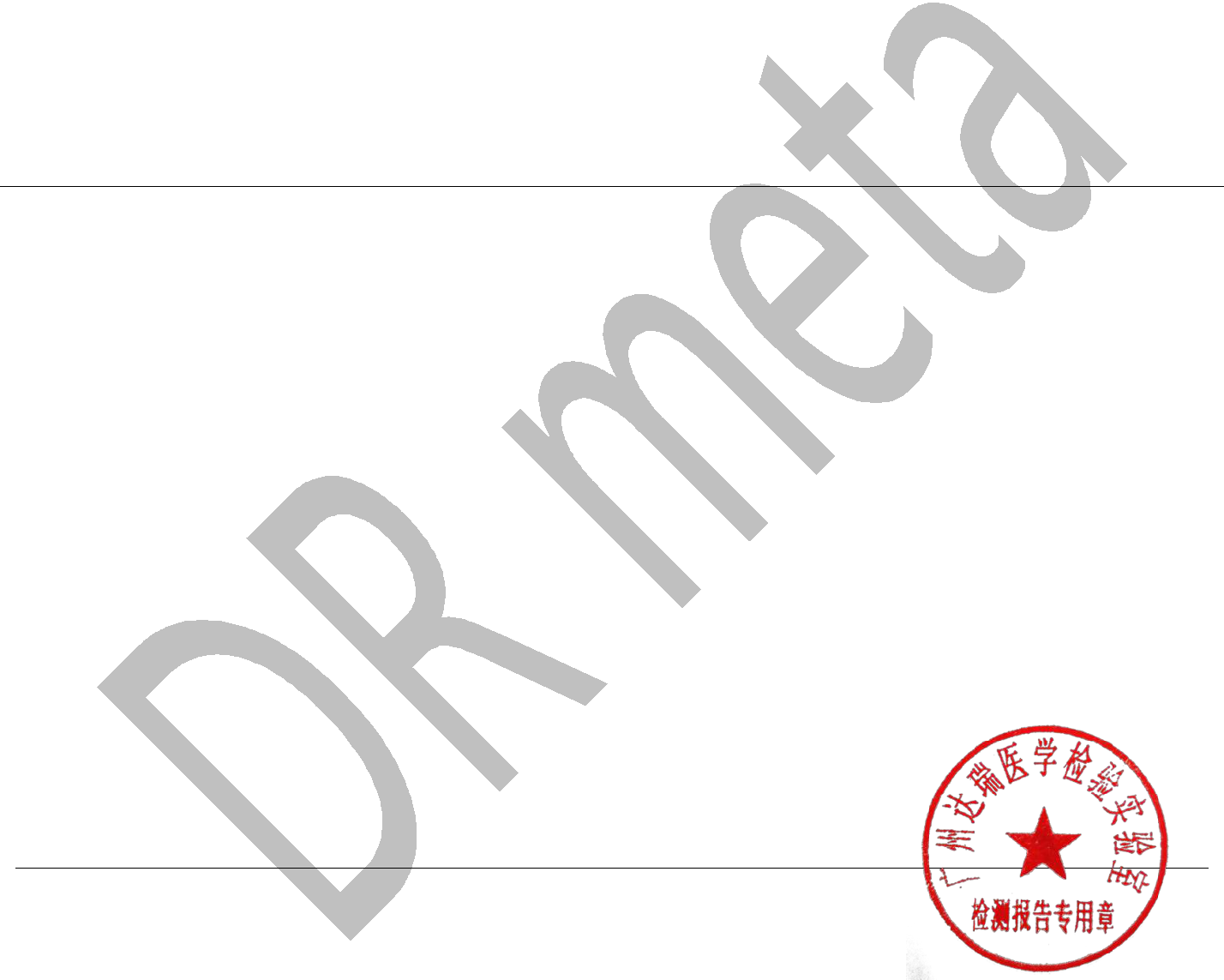


clinical diagnosis:

**Detection of pathogen information and recommendations:**

Through analysis of cerebrospinal fluid samples, it was found that the patients were mainly infected with human herpes virus type 1;

Human herpes viruses mainly invade tissues derived from the ectoderm, including skin, mucous membranes and nerve tissues. The infection sites and the diseases caused are diverse, and there is a tendency of latent infection, which seriously threatens human health. Common signs after infection are: ganglion glands, renal lymphoid tissue, lymphoid tissue febrile herpes; lip, eye, and brain infections; genital herpes varicella; herpes zoster mononucleosis, eye, kidney, brain and congenital infections Infectious mononucleosis, Burkitt lymphoma, nasopharyngeal carcinoma, infant acute eruption and other diseases such as unknown abdominal pain;

Acyclovir can be used for treatment. Specifically, the clinician is asked to conduct a comprehensive analysis based on the patient's clinical symptoms and the test results

Examiner: **** Reviewer: ****

Inspection Date: 2019.11.16 Report Date: 2019.11.17

Remarks: This report is only responsible for the samples submitted for inspection! The results are for doctor's reference only, and microorganisms below the detection limit cannot be guaranteed to be detected. If you have any questions about the test results, please contact us within 7 working days after receiving the report , Thanks for your cooperation!

Page 2 of 6

|  |  |  |  | 广州达瑞医学检验实验室 | |
| --- | --- | --- | --- | --- | --- |
| **3.detection result** | |  |  |  |  |
| 3.1 list of detected bacteria | |  |  |  |  |
|  |  |  |  |  |  |
|  |  | Category |  | Species | |
|  |  |  |  |  |  |
| Type | |  | Mapping | Mapping | |
|  | Chinese name | Latin name | Chinese name | Latin name | |
|  |  |  | sequence number | sequence number | |
|  | |  |  |  |  |
| Not  detected | |  |  |  |  |
|  |  |  |  |  |  |


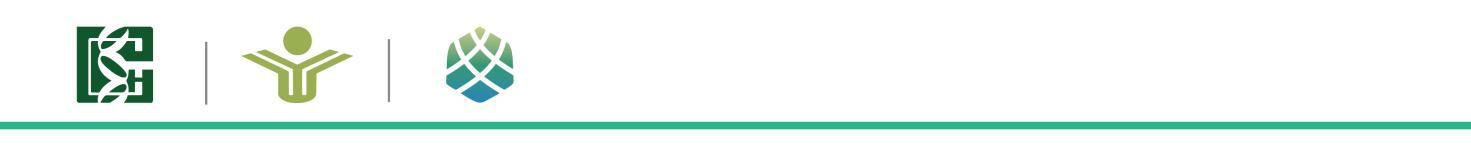

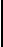


Type: G[-] stands for gram-negative bacteria, G[+] stands for gram-positive bacteria, the number of Mapping sequences refers to the number of sequences matching the pathogen. The number is related to the pathogen load, the amount of nucleic acid extraction, and the proportion of human sequence in the specimen.


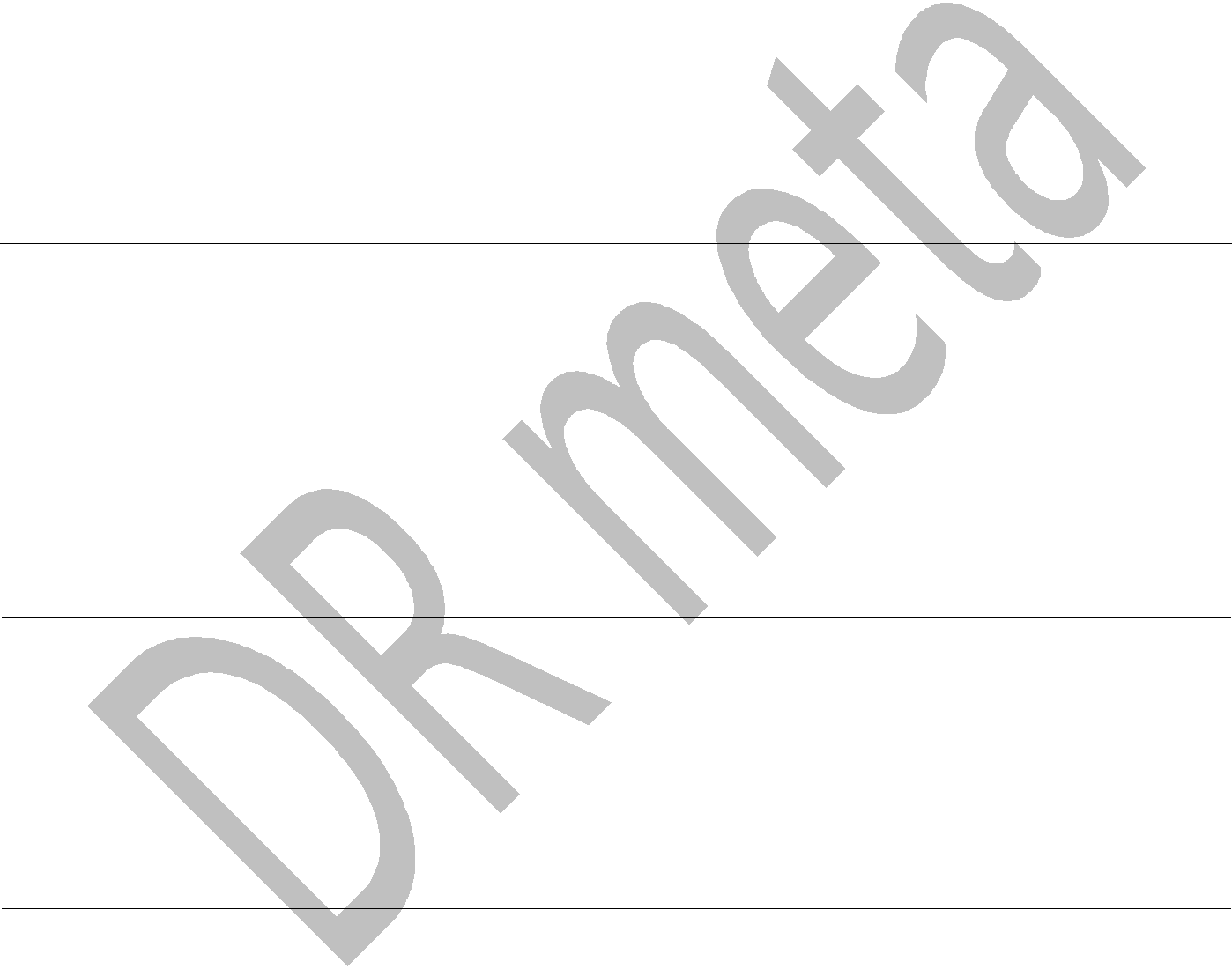


The higher the credibility of the pathogen. Genus level includes species level;

3.2 List of detected fungi

|  |  | species |  |  |
| --- | --- | --- | --- | --- |
|  |  |  |  |  |
| Type | | Mapping |  | Mapping |
|  | Chinese name | Latin name | Chinese name | Latin time |
|  |  | sequence number |  | sequence number |
|  | |  |  |  |
| Not  detected | |  |  |  |
|  |  |  |  |  |

3.3 List of detected viruses

| Chinese name |  | Latin name | Mapping sequence number |
| --- | --- | --- | --- |
|  |  |  |  |
| 人疱疹病毒 | 1 型 | *Human alphaherpesvirus 1* | 275 |
|  |  |  |  |

3.4 List of detected parasites

Chinese name Latin name Mapping Sequence number


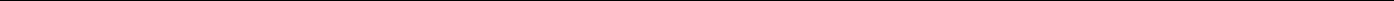


not detect
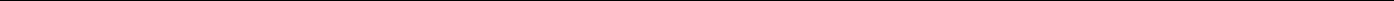


3.5 List of detected mycobacterial complexes


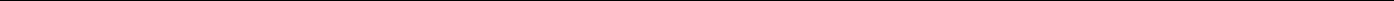


Chinese name Latin name Mapping Sequence number


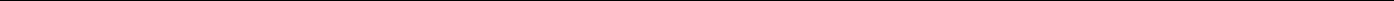


not detected


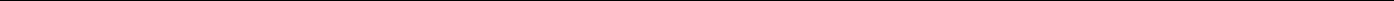


Guangzhou Darui Medical Laboratory
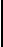


3.6 List of detected mycoplasma or chlamydia


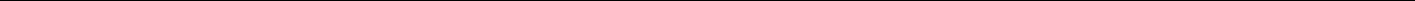


Chinese name Latin name Mapping Sequence number


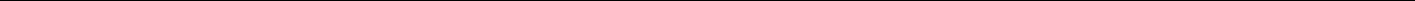


not detected


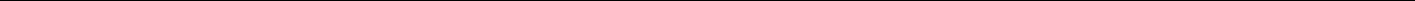


3.7 List of detected resistance genes


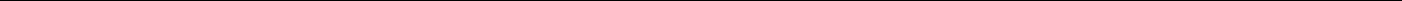


Gene comparison agreement rate (%) Antibiotics Common bacteria (species)


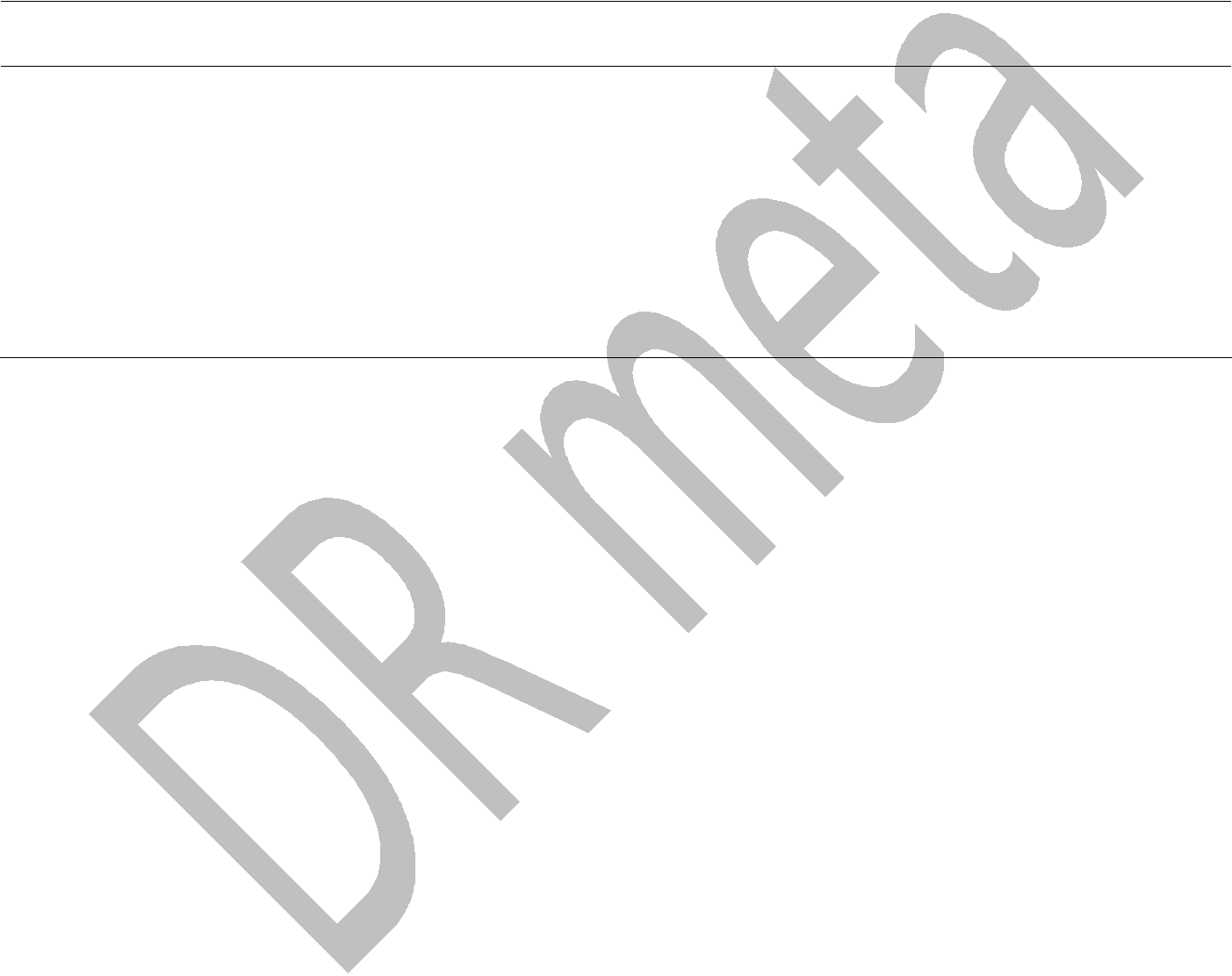


not detected

Note: Refer to the databases CARD, ARDB and UNIPROT. The alignment agreement rate is a measure of the similarity between the sequence and the reference sequence, which can be expressed by the percentage of the same base. Multi-drug resistance is an undetermined drug resistance site and is only for scientific research reference.

3.8 Appendix to the list of suspected pathogens

| Latin name | Mapping | Chinese name |  | Reference |  |
| --- | --- | --- | --- | --- | --- |
|  | sequence |  |  |  |  |
|  |  |  |  |  |  |
|  |  |  |  |  |  |
|  |  |  |  |  |  |
|  |  |  |  |  |  |
| Staphylococc  us  haemolyticus  Escherichia  coli | 6  4 |  |  | PMID: 27377762  PMID: 25726041 |  |
|  |  |  |  |  |  |

|  |  |  |
| --- | --- | --- |

Guangzhou Darui Medical Laboratory
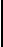


3.9 Experimental Quality Control List


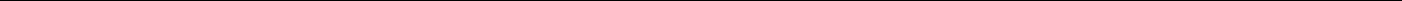


| Total sequence number checked out | Number of microbial detection sequences | Internal reference detection rate | cover degree |
| --- | --- | --- | --- |
|  |  |  |  |
| 12042600 | 1335845 | 100 | / |
|  |  |  |  |

Note:

Total number of detected sequences: the total number of nucleic acid sequences detected by high-throughput sequencing methods;

Number of microbial detection sequences: the total number of microbial nucleic acid sequences detected in the sample;


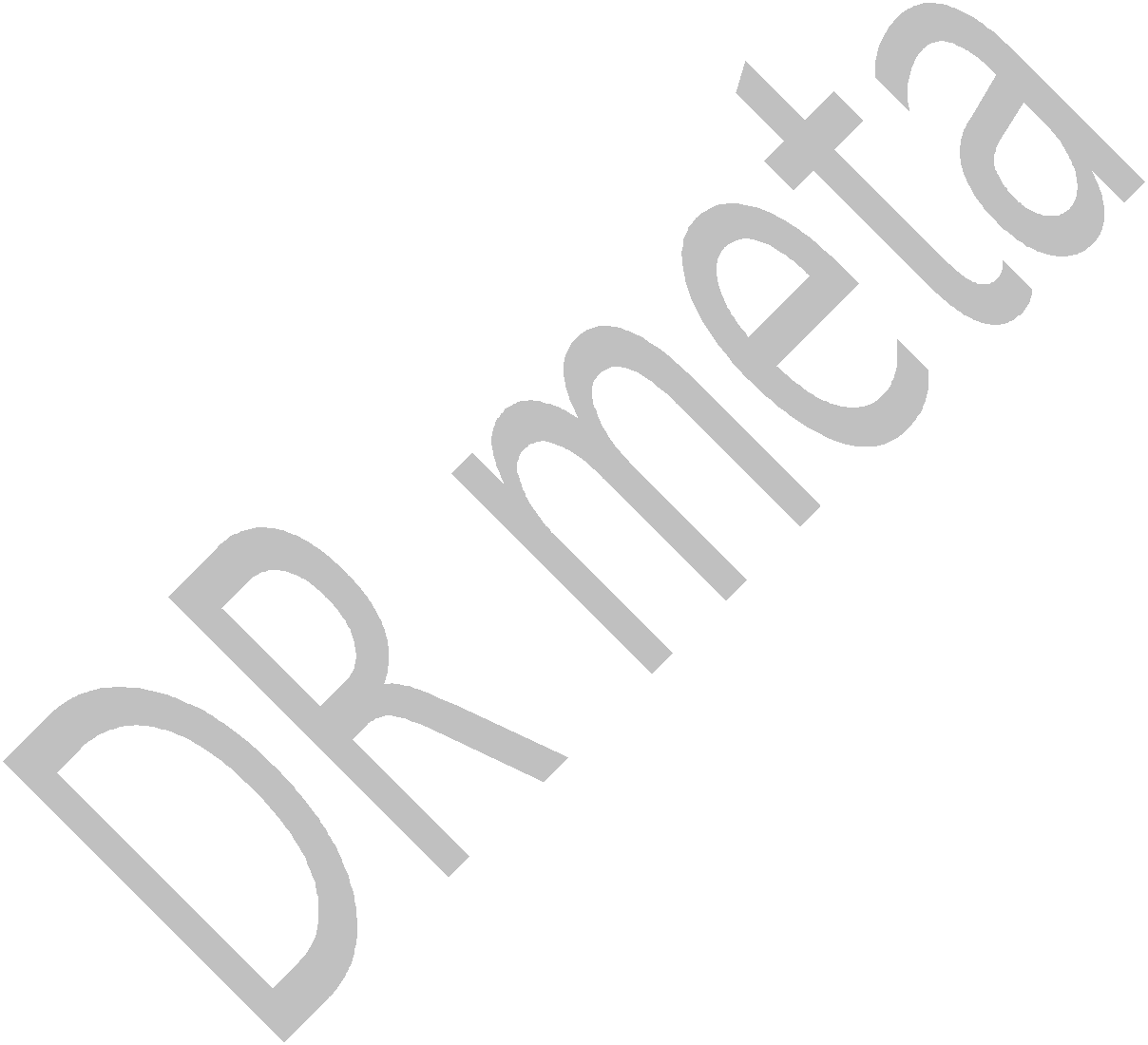


Internal reference detection rate: design a non-human, non-microbial sequence plasmid as the internal reference for the quality control of the experiment, and the comparison rate of the internal reference sequence;

Coverage: indicates the ratio of the detected nucleic acid sequence of the microorganism to the entire gene sequence of the microorganism;

Guangzhou Darui Medical Laboratory
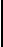


**4. References**

[1]Wilson M.R., et al. Clinical metagenomic next generation sequencing for diagnosis of infectious meningitis and encephalitis.［J］.N Engl J Med.2019

1. Fan, S., et al. Metagenomic Next-generation Sequencing of Cerebrospinal Fluid for the Diagnosis of Central Nervous System Infections: A Multicentre Prospective Study.［J］bioRxiv,2019.
2. Blauwkamp, T. A., et al., Analytical and clinical validation of a microbial cell-free DNA


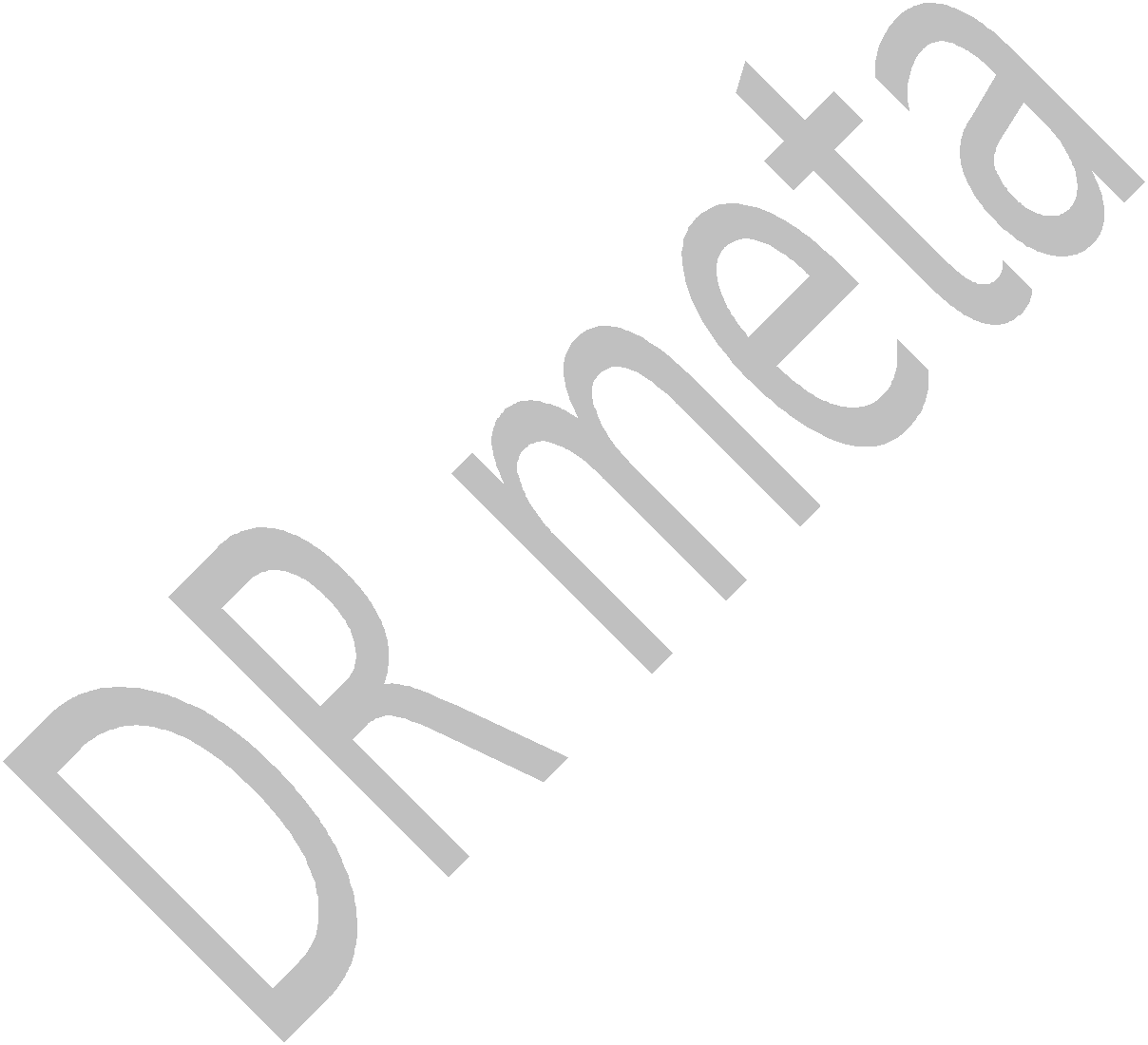


sequencing test for infectious disease.［J］Nat Microbiol, 2019, 4(4), p.663.

1. Miller, S., et al. Laboratory validation of a clinical metagenomic sequencing assay for pathogen detection in cerebrospinal fluid.［J］Genome Res, 2019.

[5]Chen H, Jiang W. Application of high-throughput sequencing in understanding human oral microbiome related with health and disease［J］. Front Microbiol, 2014, 5:508.

[6]Guan,H.,et al. Detection of virus in CSF from the cases with meningoencephalitis by next-generation sequencing.［J］Journal of neurovirology, 2016.22(2), 240-245.

[7]Lynch T,Petkau A,Knox N,et al. A primer on infectious disease bacterial genomics［J］.Clin Microbiol Rev, 2016, 29(4):881-913.

[8]Lopez-Perez M, Mirete S. Discovery of novel antibiotic resistance genes through metagenomics［J］. Recent Adv DNA Gene Seq,2014, 8(1):15-19.

[9]Blum HE. The human microbiome［J］. Adv Med Sci, 2017,62(2):414
