# Supplementary material for: A rare case of adult herpes simplex encephalitis complicated with rhabdomyolysis
Source: BMC Infect Dis. 2021 Jan 23;21:110. doi: 10.1186/s12879-021-05798-1 (PMC7825203; doi:10.1186/s12879-021-05798-1)
Supplement: Supplementary file 2 — Additional file 2. [file 12879_2021_5798_MOESM2_ESM.docx]

**Analysis report** **about one patient’s pathogenic microorganism detection**

Dear Customer:

On November 12, 2019, our company conducted a metagenomic test of pathogenic microorganisms on one cerebrospinal fluid sample, the result was herpes simplex virus type 1. The specific results are as follows, therefore the following viruses can be excluded: Dengue fever virus, Zika virus, West Nile virus, influenza A virus, H1N1 virus and scrub typhus.


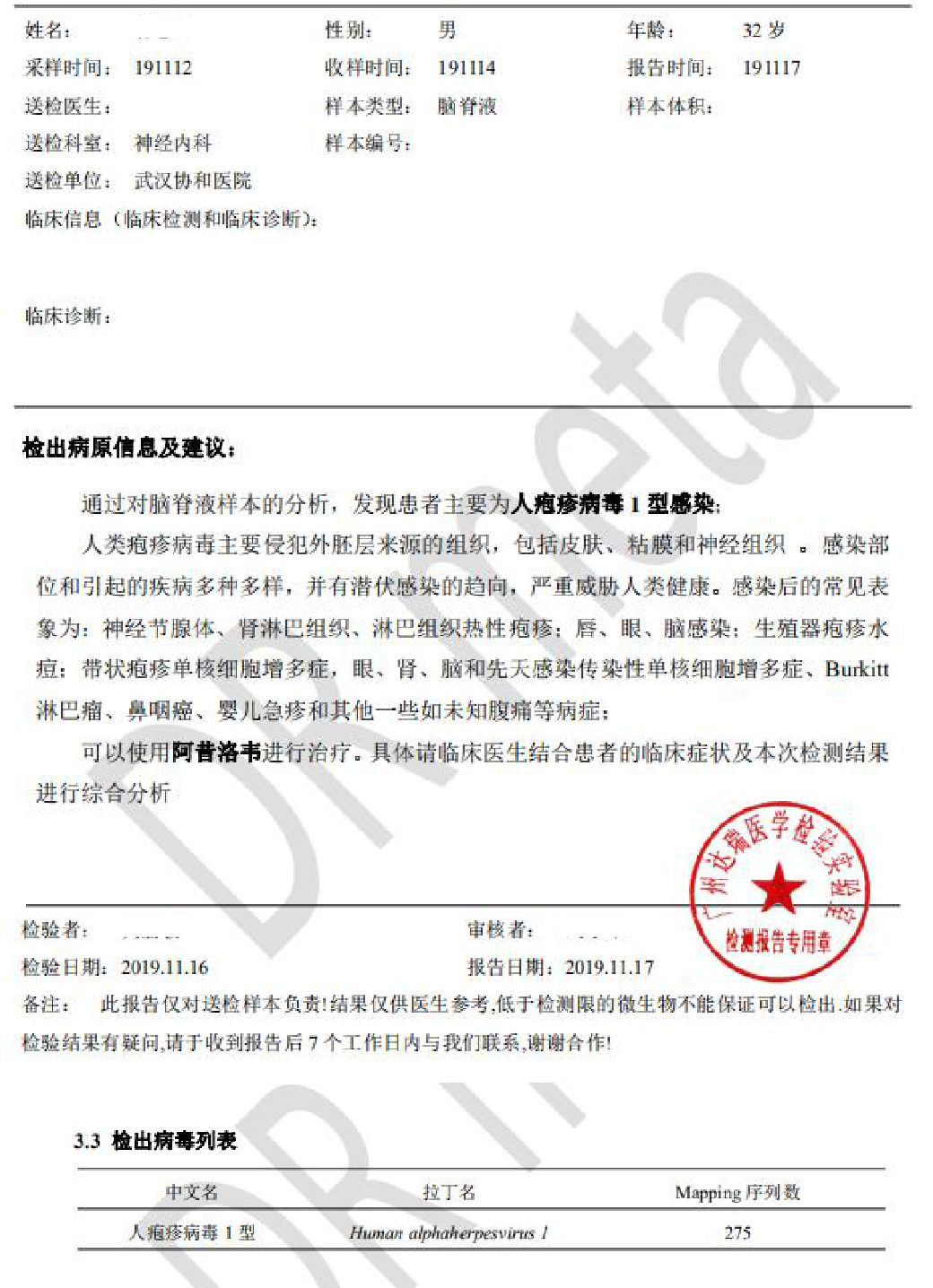

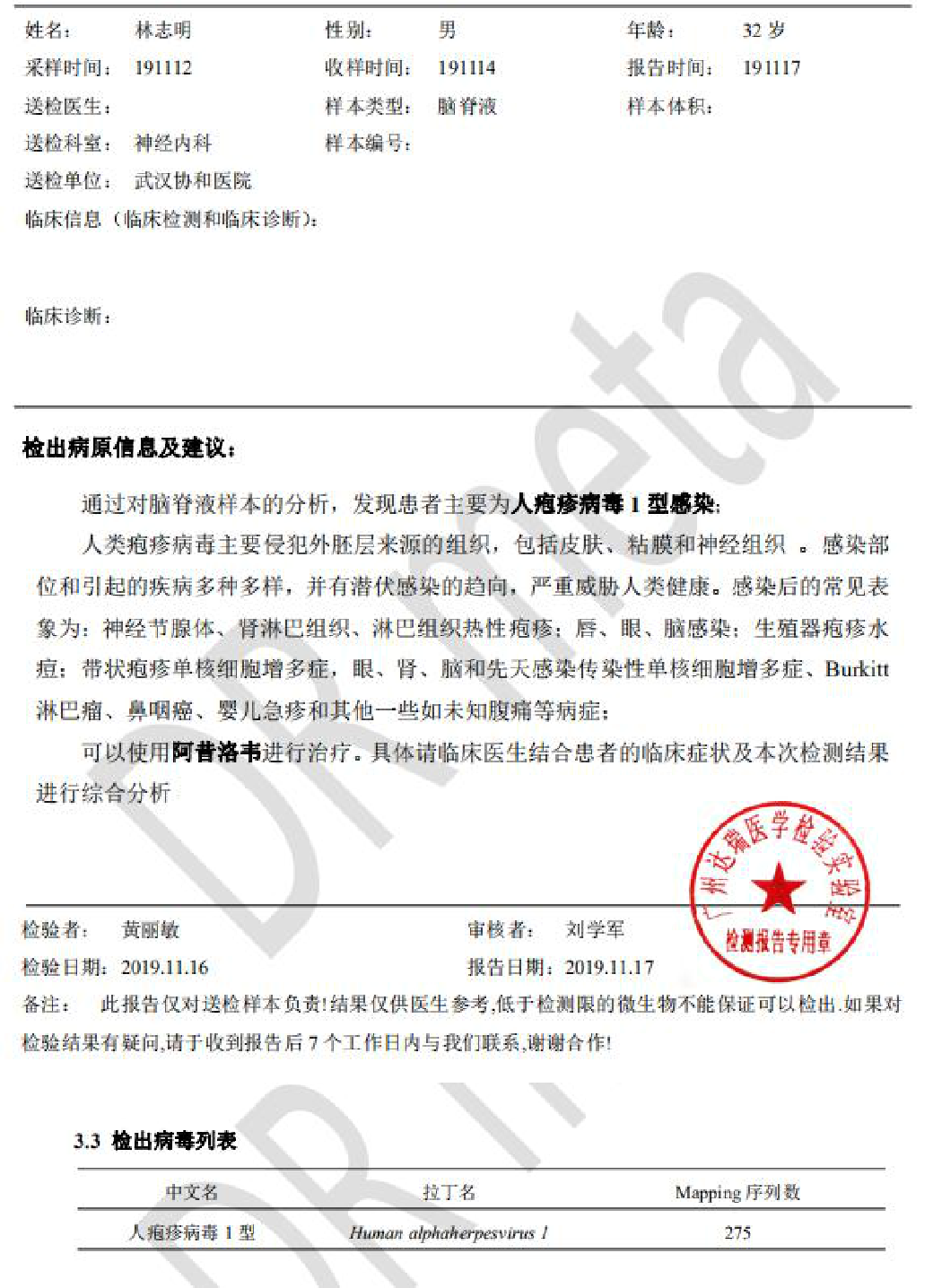


1
